# *Bacillus subtilis* as heterologous host for the secretory production of the non-ribosomal cyclodepsipeptide enniatin

**DOI:** 10.1007/s00253-014-6199-0

**Published:** 2014-11-15

**Authors:** Sophia Zobel, Jana Kumpfmüller, Roderich D. Süssmuth, Thomas Schweder

**Affiliations:** 1 Institut für Chemie, Technische Universität Berlin, Strasse des 17. Juni 124, 10623 Berlin, Germany; 2 Institut für Pharmazie, Ernst-Moritz-Arndt-Universität, Felix-Hausdorff-Strasse 3, 17489 Greifswald, Germany

**Keywords:** Heterologous expression, Iterative NRPS, Cyclodepsipeptide, *acoA* promoter, Acetoin, Metabolic engineering

## Abstract

The heterologous expression of genes or gene clusters in microbial hosts, followed by metabolic engineering of biosynthetic pathways, is key to access industrially and pharmaceutically relevant compounds in an economically affordable and sustainable manner. Therefore, platforms need to be developed, which provide tools for the controlled synthesis of bioactive compounds. The Gram-positive bacterium *Bacillus subtilis* is a promising candidate for such applications, as it is generally regarded as a safe production host, its physiology is well investigated and a variety of tools is available for its genetic manipulation. Furthermore, this industrially relevant bacterium provides a high secretory potential not only for enzymes but also for primary and secondary metabolites. In this study, we present the first heterologous expression of an eukaryotic non-ribosomal peptide synthetase gene (*esyn*) coding for the biosynthesis of the small molecule enniatin in *B. subtilis*. Enniatin is a pharmaceutically used cyclodepsipeptide for treatment of topical bacterial and fungal infections. We generated various enniatin-producing *B. subtilis* strains, allowing for either single chromosomal or plasmid-based multi-copy expression of the *esyn* cluster under the control of an acetoin-inducible promoter system. Optimization of cultivation conditions, combined with modifications of the genetic background and multi-copy plasmid-based *esyn* expression, resulted in a secretory production of enniatin B. This work presents *B. subtilis* as a suitable host for the expression of heterologous eukaryotic non-ribosomal peptide synthetases (NRPS) clusters.

**Electronic supplementary material:**

The online version of this article (doi:10.1007/s00253-014-6199-0) contains supplementary material, which is available to authorized users.

## Introduction

There is an increasing demand for new bioactive natural products, which can in many cases not be covered from natural sources (Koehn and Carter 2005). Hence, quite often, the development of fermentation processes using suitable heterologous hosts is necessary. In addition, there are many safety requirements for the industrial production of enzymes and drugs (especially for food and pharmaceutical products) which are regulated and controlled by the Food and Drug Administration (FDA) or the European Medicine Agency (EMA).

*Bacillus subtilis*, a Gram-positive, non-pathogenic strain with a generally recognized as safe (GRAS) status and qualified presumption of safety (QPS) certification can fulfil these requirements (Leuschner et al. 2010; Sietske de Boer and Diderichsen 1991). Unlike Gram-negative hosts such as *Escherichia coli*, which feature lipopolysaccharides on the outer cell membrane, *B. subtilis* lacks these endotoxins, which simplifies downstream processing (Petsch and Anspach 2000). Furthermore, using *B. subtilis* as an expression host is advantageous due to its natural ability to secrete peptides into the environment, e.g. to interact with plants or pathogens (Mongkolthanaruk 2012). It is especially noteworthy that *B. subtilis*, in contrast to *E. coli*, is a more prolific natural producer of various bioactive compounds originating from non-ribosomal peptide synthetases (NRPS) and polyketide synthases (PKS) (Stein 2005). These include the non-ribosomally synthesized lipopeptides surfactin (Arima et al. 1968; Nakano et al. 1991) and plipastatin showing antimicrobial activity (Tsuge et al. 2007), as well as the PKS/NRPS-hybrid molecule bacillaene with antibacterial effects (Butcher et al. 2007; Chen et al. 2007; Patel et al. 1995). In addition, *B. subtilis* was already successfully used as surrogate host for the engineered biosynthesis of the peptide antibiotic bacitracin from *Bacillus licheniformis* (Eppelmann et al. 2001). For the above reasons, *B. subtilis* is virtually predestined for heterologous NRPS production.

**Fig. 1:**
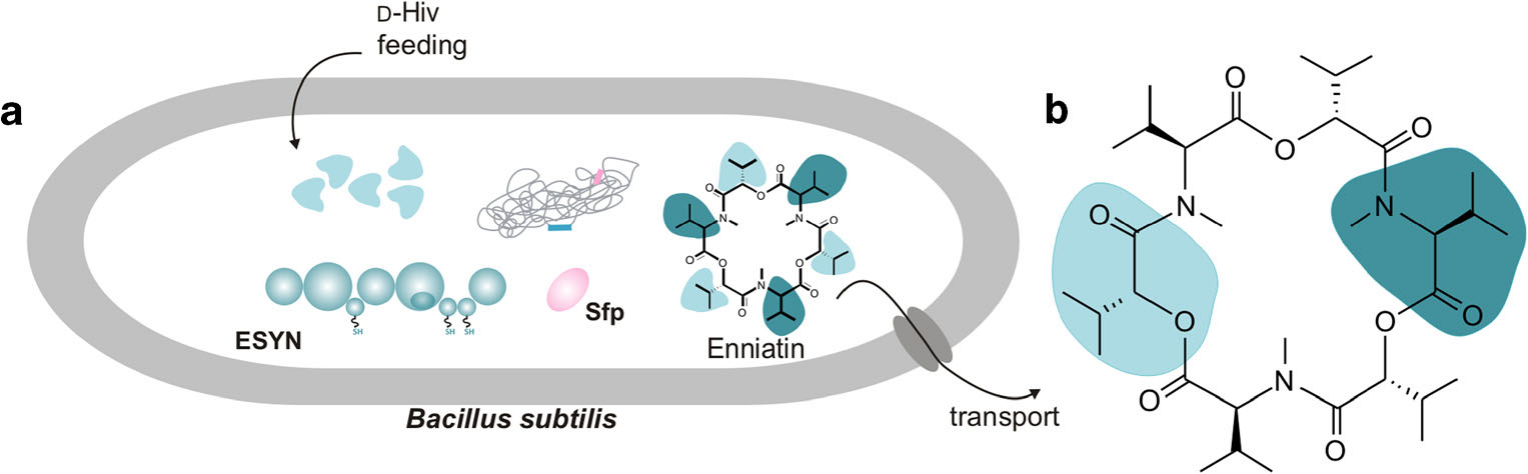
Heterologous expression of enniatin in *B. subtilis*. **a** Integrative copy of *esyn* into the genome or a high-copy plasmid under the control of an acetoin-inducible promoter (*acoA*) combined with feeding of d-Hiv facilitates synthesis of enniatin. Enniatin is synthesized non-ribosomally by the ATP-dependent non-ribosomal peptide synthetase enniatin synthetase (ESYN). **b** Structure of the cyclohexadepsipeptide enniatin composed of alternating d-hydroxyisovalerate (d-Hiv) and l-*N*-methyl-valine (l-*N*-Me-Val). The cyclic peptide is naturally produced by filamentous fungi of *Fusarium* spec

In this study, the NRPS-derived cyclohexadepsipeptide enniatin was used (Fig. 1), which is produced in a non-ribosomal fashion by various filamentous fungi such as *Fusarium* and *Verticillium* species (Süssmuth et al. 2011). These filamentous fungi are characterized by long fermentation times and are nearly insusceptible to standard procedures of genetic manipulations for production level enhancement or pathway engineering. Enniatin is well-known for a broad range of bioactivities, and it shows various antibacterial, insecticidal, antifungal, herbicidal, anthelmintic and anticancer activities (Dornetshuber et al. 2007; Kamyar et al. 2004; Kleinkauf and von Döhren 1990; Kouri et al. 2003; Pleiss et al. 1996; Süssmuth et al. 2011).

To date, 29 natural enniatin derivatives are known, which differ in their amino acid and α-hydroxy-carboxylic acid composition. A mixture of enniatin A, B and C finds application as fusafungin (Locabiosol®), a drug for treatment of upper respiratory tract infections (URTI) which shows bacteriostatic and anti-inflammatory properties against several microorganisms involved in superinfections, like *Streptococcus pneumoniae, Staphylococcus aureus, Haemophilus influenzae* and *Candida albicans* (German-Fattal 1988; Karam-Sarkis et al. 1991; Lund et al. 2004).

Enniatin is synthesized by the enniatin synthetase (ESYN), a non-ribosomal peptide synthetase which assembles the hexadepsipeptide in an iterative manner. The 347-kDa multienzyme complex consists of two modules. Each module provides a functional unit involving a condensation (C) domain, an adenylation (A) domain and a peptidyl carrier protein (PCP) domain (Glinski et al. 2002; Pieper et al. 1995). The A domains recognize their l-configured amino acid and d-α-hydroxy carboxylic acid substrate and activate them in an ATP-dependent manner by adenylation at the carboxy group to yield acyl monoadenylates. The activated substrates are then transferred to a PCP domain, posttranslationally modified with a CoA arm, which is the acceptor for thiolation with the substrate. This PCP domain is phosphopantheteinylated by a phosphopantetheine transferase (PPTase). Intermediates that are covalently bound to the phosphopantetheine prosthetic group of the PCP domain are subsequently presented to the C domain, located upstream or downstream, responsible for the formation of the corresponding ester or amide bond between two activated substrates (Billich and Zocher 1988). An intermediate step is *N*-methylation of the l-amino acid by a methylation domain (MT domain). Emerging dipeptidoles are then temporarily stored at a so-called waiting position operated by a third PCP domain until the next dipeptidole subunit is formed and can be loaded onto the last PCP domain forming a 4-mer peptidole (Süssmuth et al. 2011). Upon availability of the third and last dipeptidole, the synthesis is completed by a last condensation and a final cyclisation step which takes place to release the active cyclohexadepsipeptide.

In this work, we established the first heterologous expression of a fungal non-ribosomal peptide synthetase and synthesis of the corresponding peptide enniatin in *B. subtilis*. In this study, the *esyn* gene encoding the enniatin synthetase (ESYN) from *Fusarium oxysporum* (Zocher et al. 1982) was used as a model for the heterologous expression of a NRPS cluster in *B. subtilis* under control of a glucose-repressed and acetoin-inducible promoter system (Ali et al. 2001; Kabisch et al. 2013b; Silbersack et al. 2006). In order to optimize the enniatin production in this host, several cultivation conditions and the deletion of potential competing gene clusters for native secondary metabolites were addressed.

## Materials and methods

### Cloning

Unless stated otherwise, all chemicals were purchased from Roth (Karlsruhe, Germany) at the highest purity available and were used without further purification. *B. subtilis* 168 (NCBI AL009126) was used as model production strain in this study. All cloning procedures were carried out in *E. coli* DH10B (Invitrogen, Darmstadt, Germany) [F-endA1 recA1 galE15 galK16 nupG rpsL ΔlacX74 Φ80lacZΔM15 araD139 Δ(ara, leu)7697 mcrA Δ(mrr-hsdRMS-mcrBC) λ-]. All plasmids used in this study are listed in Table 1. All strains used and constructed in this study are summarized in Table 2. Restriction enzymes and other DNA-modifying enzymes were used as specified by the supplier (New England Biolabs, Frankfurt, Germany). Oligonucleotides (Table S1) were synthesized and provided by Life Technologies (Darmstadt, Germany). PCR products were purified with the High Pure PCR Product Purification Kit (Roche, Mannheim, Germany). Plasmid isolation was performed using the High Pure Plasmid Isolation Kit (Roche, Mannheim, Germany). For gel extraction, the QIAquick Gel Extraction Kit from Qiagen (Hilden, Germany) was used. Recombinant *B. subtilis* strains were verified by colony PCR as previously described (Kumpfmüller et al. 2013). All plasmid constructs and chromosomal integrations (s. supporting online material) were verified by sequencing carried out by Eurofins Genomics (Ebersberg, Germany).

**Table 1:** Plasmids used in this study

Plasmid	Function	Reference
Fosmid F9D10	Fosmid carrying wild-type *esyn* gene cluster	Fosmid library of *F. oxysporum* ETH 1536, ESyn ACCN KP000028
pAMY-Kan	Backbone for *srfA* deletion plasmid with Kan^R^	Kumpfmüller, unpublished results
pAMY-lox-SSS	Integration of genes into the *amyE* locus with *lox*-SSS-cassette	Kumpfmüller et al. 2013
pAMYSSE	Integration of genes into the *amyE* locus with SSE-cassette	Kabisch et al. 2013a
pAMY-SSS	Integration of genes into the *amyE* locus with SSS-cassette	Kumpfmüller et al. 2013
pBB1366	Integration of genes in *sacA* locus with Cm^R^	Middleton and Hofmeister 2004
pDGICZ	Source of *cre* operon and Zeo^R^	Yan et al. 2008
pJET-lox-SSS	Source of *lox*-SSS-cassette	Kumpfmüller et al. 2013
pJK45	Integration of *comS* operon (*P*_*spac*_) into *sacA* locus with Cm^R^	This study
pJK64	Reconstitution of genetic *sfp* defect with SSS-cassette	This study
pJK64a	Reconstitution of genetic *sfp* defect with *lox*-SSS-cassette	This study
pJK93	Deletion of *srfA* operon with remaining Kan^R^	This study
pJK166	Integration of *P*_*acoA*_ *-esyn-T*_*T7*_ into *amyE* locus	This study
pJK179	Deletion of *pksX* operon	This study
pJK191	Deletion of *srfA* operon	This study
pJK195	Integration of *cre* operon (*P*_*xylA*_) into *sacA* locus with Zeo^R^	This study
pJK196	Integration of *comS* operon (*P*_*spac*_) and *cre* operon (*P*_*xylA*_) into *sacA* locus with Zeo^R^	This study
pJK205	Insertion in *lytC* locus	This study
pJK209	Deletion of *spoIIGA*	This study
pJK226	Deletion of restriction and modification system (RM)	This study
pJK210	Integration of genes in *sacA* locus with Spec^R^	This study
pJK255	High-copy expression of *P*_*acoA*_ *-esyn-T*_*T7*_	This study
pJK256	Substitution of *comS* operon (*P*_*spac*_) and *cre* operon (*P*_*xylA*_) with Spec^R^	This study
pKE19	Source of *srfA* 5′-region with reconstituted *comS* gene	Eppelmann et al. 2001
pKE27	Source of *comS* operon (*P*_*spac*_)	Eppelmann et al. 2001
pLytC	Backbone for *lytC* deletion plasmid with SSC-cassette	Kabisch et al. 2013b
pMSE3	High-copy *E. coli*/*B. subtilis* shuttle vector with Kan^R^	Silbersack et al. 2006
pSigL	Backbone for *pksX* deletion plasmid with SSE-cassette	Kabisch et al. 2013b
pSpoIIGA	Backbone for *spoIIGA* deletion plasmid with SSS-cassette	Kabisch et al. 2013b
pX	Source of the *xylA*-promoter and *xylR* gene	Kim et al. 1996

*CmR* chloramphenicol resistance cassette, *EryR* erythromycin resistance cassette, *KanR* kanamycin resistance cassette, *SpecR* spectinomycin resistance cassette, *ZeoR* Zeocin resistance cassette, *ss* six-site, *lox72* lox72 site, *SSS* SpecR flanked by two ss, *SSE* EryR flanked by two ss, *SSC* CmR flanked by two ss, *lox-SSS* SSS surrounded by a *lox71* and *lox66* site

**Table 2:** Strains used in this study

Strain	Relevant genotype	Reference
*B. subtilis* 168	Wild type, sfp^0^	Zeigler et al. 2008
*B. subtilis* ATCC 6051HGW	Wild type, sfp^+^	Kabisch et al. 2013b
*B. subtilis* JK3	*ΔsacA::(Cm* ^*R*^ *, P*_*spac*_ *-comS, lacI)*	This study
*B. subtilis* JK13	*ΔsacA::(Zeo* ^*R*^ *, P*_*spac*_ *-comS, lacI, P*_*xylA*_ *-cre, xylR)*	This study
*B. subtilis* JK28	*BsJK13 + sfp* ^*+*^ *::lox72*	This study
*B. subtilis* JK46	*BsJK28 + RM::lox72*	This study
*B. subtilis* JK75	*BsJK46 + ΔsrfA::lox72*	This study
*B. subtilis* JK76	*BsJK75 + ΔpksX::lox72*	This study
*B. subtilis* JK77	*BsJK76 + ΔlytC::lox72*	This study
*B. subtilis* JK78	*BsJK77 + ΔspoIIGA::lox72*	This study
*B. subtilis* JK105	*BsJK78 + ΔamyE::lox72*	This study
*B. subtilis* JK106	*BsJK105 + ΔsacA::Spec* ^*R*^	This study
*B. subtilis* JK106 (pJK255)	High-copy expression strain	This study
*B. subtilis* SZ2	*BsJK3 + ΔamyE::(P*_*acoA*_ *-esyn-T*_*T7*_ *, ss)*	This study
*B. subtilis* SZ4	*BsSZ2 + sfp* ^*+*^ *::ss*	This study
*B. subtilis* SZ5	*BsSZ4 + ΔsacA::(Zeo* ^*R*^ *, P*_*spac*_ *-comS, lacI, P*_*xylA*_ *-cre, xylR)*	This study
*B. subtilis* SZ6	*BsSZ5 + ΔlytC::lox72*	This study
*B. subtilis* SZ7	*BsSZ6 + ΔspoIIGA::lox72*	This study
*B. subtilis* SZ8	*BsSZ7 + ΔsacA::Spec* ^*R*^	This study
*B. subtilis* SZ9	*BsSZ7 + ΔsrfA::lox72*	This study
*B. subtilis* SZ10	*BsSZ9 + ΔsacA::Spec* ^*R*^	This study
*B. subtilis* SZ11	*BsSZ9 + ΔpksX::lox72*	This study
*B. subtilis* SZ12	*BsSZ11 + ΔsacA::Spec* ^*R*^	This study

*CmR* chloramphenicol resistance cassette, *SpecR* spectinomycin resistance cassette, *ZeoR* Zeocin resistance cassette, *ss* six-site, *lox72 lox72* site

### Media and cultivation

For the cultivation of *B. subtilis* strains, a 20-mL pre-culture was inoculated with a cryo-culture in LB medium (10 g/L tryptone, 5 g/L yeast extract, 5 g/L NaCl) supplemented with the corresponding antibiotic. After incubation for 16 h at 37 °C in 50 mL super broth (SB) medium (containing 95 % solution 1 buffered with 5 % of solution 2; solution 1: 32 g/L tryptone, 20 g/L yeast extract, 5 g/L NaCl, pH 7; solution 2: 12 g/L Na_2_HPO_4_, 6 g/L KH_2_PO_4_, 6 g/L NH_4_Cl, 6 mg/L CaCl_2,_ pH 7), a second pre-culture was inoculated with 1:100 (*v*/*v*) and 0.1 % acetoin (*v*/*v*) together with antibiotics and incubated at 37 °C to optical density at 600 nm (OD_600 nm_) = 1.0. For the main culture, 100-mL shaking flasks with three baffles were used containing 20 mL of SB medium supplemented with antibiotics and 0.1 % acetoin (*v*/*v*). The medium was inoculated at OD_600 nm_ = 0.1 and incubated for 1.5 h at 37 °C to reach the exponential phase at OD_600 nm_ = 0.6. Subsequently, the cultures were fed with appropriate concentrations of 5 mm d-Hiv and incubated at 18 °C for 48 h under shaking conditions with 200 rpm.

### Extraction

After cultivation and harvesting of cells from 20-mL cultures, the supernatant was extracted with an equal volume of ethyl acetate and agitated for 1 h at room temperature. The cell pellet was extracted with 5 mL methanol and sonicated for 5 min. After centrifugation, the solvent was removed and evaporated under vacuum. For mass spectrometric analysis, the extracts were dissolved in 200 μL HPLC-grade MeOH.

### HPLC-ESI-mass spectrometry

All measurements for the analysis of crude extracts of pellet and supernatant were performed using an Agilent UHPLC 1290 Infinity-Series system containing an Eclipse Plus C18 column (2.1 × 50 mm) coupled to an ESI-Triple-Quadrupol mass spectrometer (6460 Series, Agilent Technologies, Waldbronn, Germany). For chromatographic separation, a mobile phase H_2_O (solvent A)/ACN (solvent B) each with 0.1 % formic acid (*v*/*v*) was used. The gradient started at 5 % solvent B to reach 100 % in 4 min and was held constant for 3 min at 100 % solvent B. For MS scan, MS^2^ and multiple reaction monitoring (MRM) analysis of enniatin with an exact mass [M] = 639.4095 Da, all measurements were performed in the positive mode. The three most abundant fragments of MS^2^ experiments were used for quantification in MRM by fragmentation of precursor ion *m/z* 640 as well the characteristic mass transitions *m/z* 527.4 as quantifier and *m/z * 427.3 and *m/z* 196.2 as qualifier ion. For relative quantification, we used a calibration curve of enniatin B and integrated the measured peak areas accordingly. Calibration curves comprising five concentrations in the range of 0.586-9.375 μg/mL were measured before and after the measurements of enniatin B from *B. subtilis* extracts (Fig. S1).

### Software

Graphs were created using the graphical and statistical program R with the ggplot2 package (Wickham 2009). For box plots, the whiskers extend from the hinge to the highest and lowest value, respectively, that is within 1.5 × IQR of the hinge, where IQR is the inter-quartile range. Data beyond the end of the whiskers are outliers and plotted as points.

## Results

Codon usage may exert an influence on gene expression (Nocon et al. 2014; Samant et al. 2014). Therefore, we first performed a bioinformatics analysis of the *esyn* gene. A comparison of the codon usage of the *F. oxysporum esyn* sequence with the average codon usage of dedicated host *B. subtilis* 168 showed a mean deviation of about 35 % (Codon Usage Database, Kazusa; graphical codon usage analyser, Fig. S2). This analysis indicates that there is no significant codon bias which could influence the *esyn* expression in *B. subtilis*.

The plasmid construction for the chromosomal integration of the *esyn* gene, encoding the enniatin synthetase, was done via a modified protocol using the Red/ET recombination system (Zhang et al. 1998). Due to this method that is based on a crossover step between a targeting vector containing homologous regions and the target sequence (fosmid F9D10 carrying the natural *esyn* gene), amplification of the entire coding sequence (approx. 10 kb) was not necessary. The resulting plasmid was designated pJK166. A detailed description of all plasmid and strain constructions can be found in the supplementary material.

The plasmid pJK166 was used to chromosomally integrate the *esyn* gene cluster under control of the *acoA* promoter into the *amyE* locus of *B. subtilis* JK3. This integration event was confirmed by colony PCR and an amylase-negative phenotype. Finally, one of the positive clones was chosen for marker removal (resulting in *B. subtilis* SZ2) followed by chromosomal integration of an intact *sfp* gene by using the pJK64 plasmid. Obtained colonies were checked for haemolytic activity on sheep blood agar plates indicating restored surfactin production and hence PPTase activity due to a functional *sfp* gene. After marker removal, one colony of the final strain *B. subtilis* SZ4 was chosen for genomic DNA isolation and the chromosomal reconstitution of the *sfp* gene and the functionality of the *esyn* cluster were identified by means of PCR followed by DNA sequencing.

The selected *esyn*-encoding strain *B. subtilis* BsSZ4 was cultivated at 37 °C in SB medium with 0.1 % acetoin as the inducer. Supernatant and biomass were searched for the presence of enniatin (enniatin B is the main product of ESYN and, for reasons of simplicity, named enniatin) by means of HPLC-ESI-mass spectrometry. This analytics rendered the detection of enniatin adduct ions [M + H]^+^ = 640.4 Da, [M + NH_4_]^+^ = 657.4 Da and [M + Na]^+^ = 662.4 Da in the supernatant, whereas only minute amounts were found in the biomass (Fig. S3). Due to this repeated outcome of all fermentation runs, the subsequent analyses were mainly performed with culture supernatants.

The identity of enniatin was further corroborated by means of HPLC/ESI-MS^2^ experiments (Fig. S4) upon identification of characteristic masses of di- and tetrapeptidoles from fragmentation reactions at the less stable ester bonds. In the analytical assignment of secondary metabolites, we further observed the molecular masses of bacillaene and surfactin, previously identified from *Bacillus* species (Chen et al. 2007, Fig. S5). In MRM measurements, minor amounts of enniatin (∼0.4 μg/L) were detected.

**Fig. 2:**
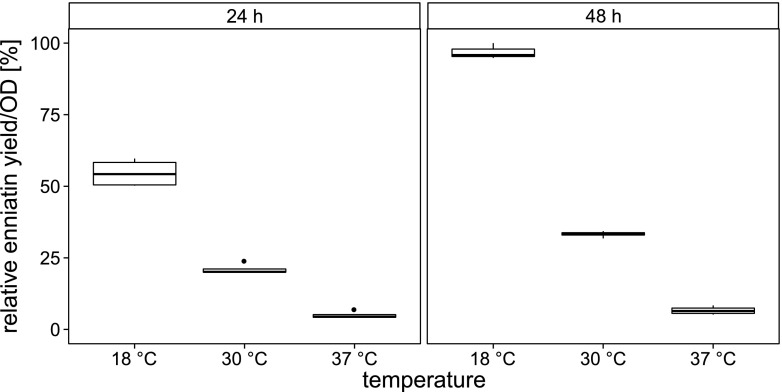
Increased enniatin production by shifting the cultivation condition to lower temperatures. Cultures of BsSZ4 were grown under the same conditions with different temperatures (18, 30 and 37 °C). *N* = 4

In order to improve the enniatin production, variations in the cultivation conditions like temperature, concentration of the inductor acetoin and availability of required precursors in the form of d-hydroxyisovalerate (d-Hiv) and l-valine (l-Val) were tested. To minimize the metabolic burden for cells expressing a multi-enzyme of that size like enniatin synthetase (Pfeifer and Khosla 2001), we lowered the incubation temperature to slow down recombinant protein synthesis (Schein 1989). On the one hand, we chose 30 °C as well-known growth temperature of the ubiquitous soil bacterium *B. subtilis*. On the other hand, according to expression results, we defined 18 °C as reasonable compromise between bacterial growth and protein expression. Remarkably, lowering the temperature resulted in higher enniatin synthesis (Fig. 2) compared to 37 or 30 °C. Furthermore, also a doubling of the cultivation duration led to a significantly increased enniatin production.

**Fig. 3:**
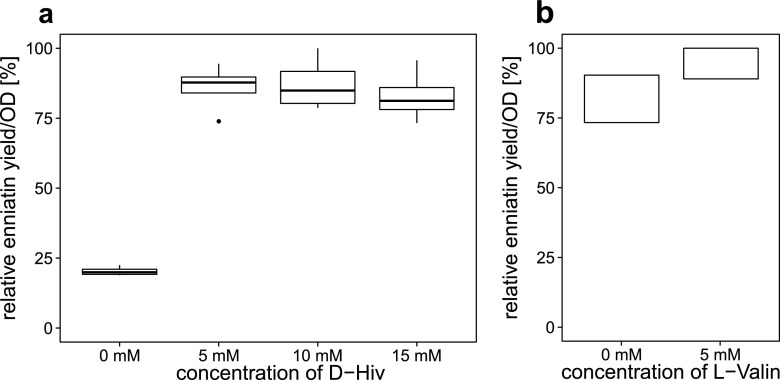
Influence of precursor feeding on enniatin production. **a** Supplementation with d-hydroxy isovalerate (d-Hiv): absence of d-Hiv in the medium renders low enniatin yields in the supernatant. A concentration of 5 mm triples enniatin production. Higher amounts of d-Hiv have no effect. *N* = 4. **b** Supplementation with l-Val: in order to compensate l-Val consumption during protein and peptide synthesis, 5 mm l-Val were fed together with 5 mm d-Hiv to the cultures and raised enniatin production by >10 %. *N* = 2; thus, no median is shown

Enniatin production of a *Bacillus* strain carrying the *esyn* gene and displaying only a basal synthesis of d-Hiv is dependent on supplementation of this building block to the medium, and this strain is therefore a dependent host. Based on the absence of the native fungal d-hydroxyisovalerate dehydrogenase, which catalyzes the transformation of 2-ketoisovalerate (2-Kiv) to d-Hiv, the substrate of ESYN (Lee et al. 1992) is missing in the recombinant host *B. subtilis*. Low production of enniatin was observed without supplementation, indicating that there is some d-Hiv available, probably in the undefined complex media ingredients like yeast extract. Upon addition of various concentrations of d-Hiv, we were able to enhance enniatin production significantly, although the overall feeding of d-Hiv above 5 mm did not further influence enniatin yields (Fig. 3a). In addition, requirements for l-Val in primary metabolism, i.e. ribosomal synthesis of l-Val-rich ESYN (7.8 %) and enniatin was compensated by feeding l-Val. Hence, adding 5 mm of l-Val to the cultures after induction boosted the enniatin production of BsSZ4 by at least another 10 % as determined by HPLC-MS (Fig. 3b).

In further experiments, we tested the inductor acetoin in different concentrations in the range of 0.1, 0.5, 1.0 and 1.5 % (*v*/*v*). We observed an increase of 10–20 % enniatin production using 1.0 and 1.5 % acetoin for induction of ESYN expression (Fig. S6). In contrast to the concentration range of 0.1 and 0.5 % final acetoin, higher amounts of acetoin (1.0 and 1.5 %) are coupled to increased cell lysis (data not shown). Therefore, for further experiments and optimization regarding the genetic background of the enniatin producing strain BsSZ4, we chose 0.1 % acetoin, 5 mm d-Hiv, no additional l-Val (because no stringent necessity for enniatin production) and 48 h cultivation time at 18 °C.

**Fig. 4:**
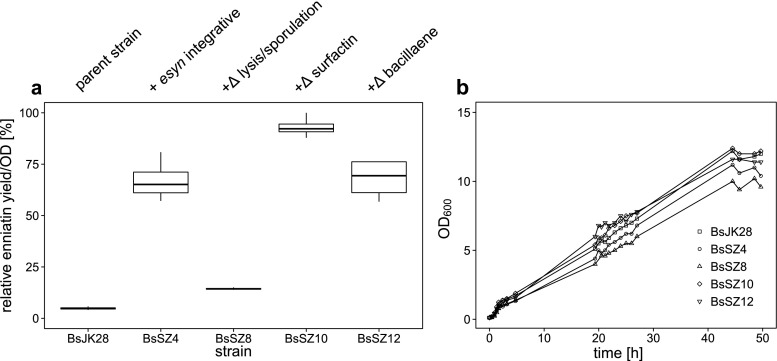
Engineering of the genetic background of the enniatin producing strain *B. subtilis* BsSZ4. **a** Quantitative MRM analysis of secreted enniatin of modified *B. subtilis* strains after cultivation for 48 h in SB medium. *N* = 4. **b** Growth curves (OD_600__nm_) of engineered *B. subtilis* strains during cultivation for 48 h in SB medium. *N* = 1. BsJK28, parental strain; BsSZ4, + *esyn*; BsSZ8, with a *lytC/spoIIGA* inactivation; BsSZ10, deletion of the surfactin cluster; BsSZ12, with an additional inactivation of bacillaene synthesis

As mentioned above, the production of enniatin is accompanied by a surfactin and bacillaene synthesis, which is also reflected by a high level of the proteins SrfAA and SrfAB visible in a SDS-PAGE (Fig. S7) and confirmed by tryptic digest of protein bands in LC-ESI-MS^2^ measurements (data not shown). Since synthesis of unwanted protein appears as an additional metabolic burden, we consequently considered inactivation of these clusters by gene deletion, which ultimately could save equivalents of energy like ATP for substrate activation, coenzyme A, precursors, cofactors and Sfp capacity for posttranslational phosphopantetheinylation of multienzymes. In order to optimize growth behaviour, we first inactivated genes involved in autolysis (*lytC*) and sporulation (*spoIIGA*). Therefore, we stepwise engineered the BsSZ4 strain with an integrated single copy of *esyn* and observed a surprisingly significant reduced enniatin synthesis in the resulting BsSZ8 strain (Fig. 4a). An additional inactivation of the surfactin gene cluster in BsSZ10 compensates these losses and leads to an increase of around 20 % of enniatin yield. As opposed to this, an additional inactivation of the bacillaene cluster in BsSZ12 surprisingly led to significantly decreased enniatin production (25 %) compared to BsSZ10.

The genetically modified strains revealed different growth behaviours (Fig. 4b). Particularly the sporulation and *lytC* deficient BsSZ8 strain showed decreased optical densities in the time course measurements. The highest OD_600 nm_ of 12.2 was observed for the best enniatin producing strain BsSZ10 being deficient in surfactin synthesis closely followed by the parental strain BsJK28 (OD_600 nm_ = 11.4). The lowest production of enniatin by BsSZ8 (with a *lytC/spoIIGA* inactivation) correlates with the lowest cell density of OD_600 nm_ = 9.6 in total. In the wake of BsSZ10 under optimized cultivation conditions together with modification of the genetic background, we obtained a secretory production of 4.7 μg/L enniatin.

**Fig. 5:**
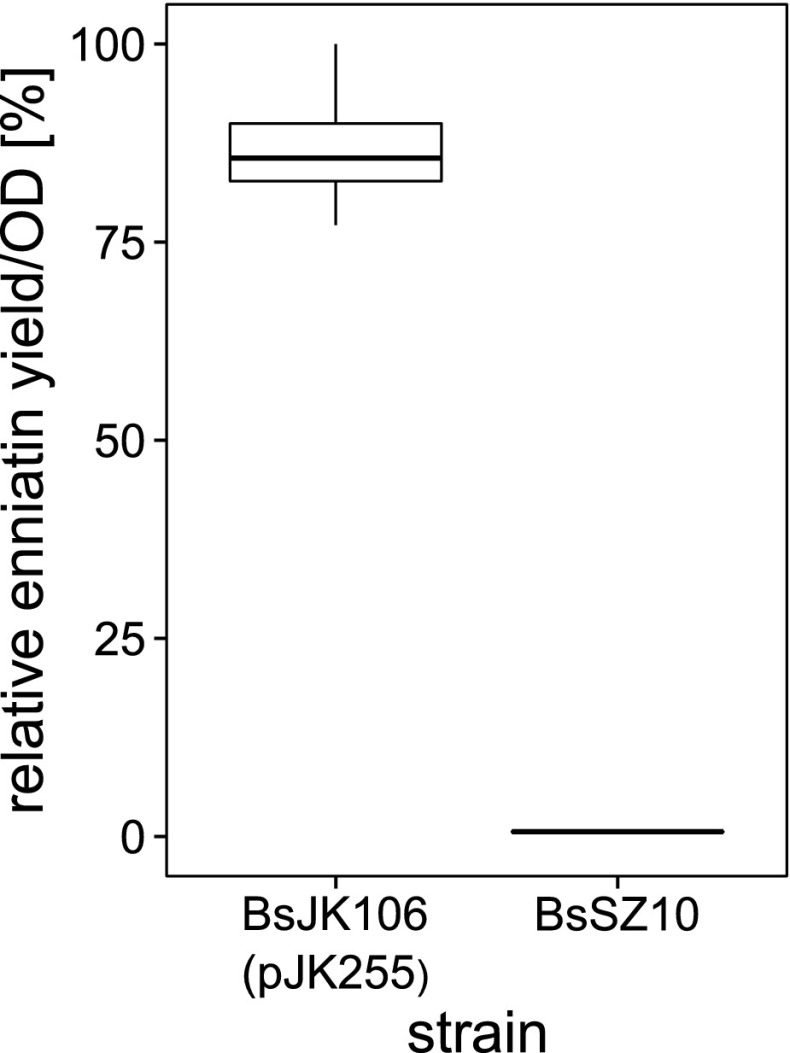
Extra-chromosomal multi-copy expression of *esyn* in *B. subtilis*. Expression of the *esyn* gene on the multi-copy plasmid pJK255 (with ∼200 copies) in BsJK106 in comparison to BsSZ10 leads to 130-fold increased enniatin level in the supernatant. *N* = 4

We furthermore tested the effect of the cloning of the *esyn* cluster into the high-copy plasmid pMSE3. The resulting strain BsJK106 (pJK255), which possesses approximately 200 copies of the *esyn* gene (Fig. S8), produced the highest yield of enniatin (Fig. 5). We quantified the extracts of supernatant of BsJK106 cultures in comparison to an external enniatin standard and measured yields of 1.1 mg/L enniatin under optimized cultivation conditions. An additional gene copy of the positive transcription factors *sigL* and *acoR* (Ali et al. 2001; Kabisch et al. 2013b) did not lead to a further enhanced enniatin production (data not shown).

## Discussion

This contribution reports on a cellular system for heterologous expression of a fungal, non-ribosomally synthesized peptide based on the Gram-positive bacterium *B. subtilis*. To assess the suitability of this expression host for heterologous NRPS expression, the non-ribosomal peptide synthetase-encoding gene *esyn* from the fungus *F. oxysporum* was used. This choice was based on the natural capacity of *B. subtilis* for the overproduction of the non-ribosomally synthesized lipopeptides surfactin and plipastatin or the PKS/NRPS-hybrid molecule bacillaene. As shown by the results of this study, in *B. subtilis*, enniatin is actively exported, probably using a native secondary metabolite transporter system, whereas *esyn* expression in the Gram-negative expression host *E. coli* leads to an exclusive intracellular accumulation of enniatin (data not shown). Similar intracellular accumulation of metabolites was observed in previous studies on heterologous expression in *E. coli* regarding the homologous cyclooligomer depsipeptide synthetases, e.g. beauvericin synthetase of *Beauveria bassiana* (Matthes et al. 2012) or valinomycin synthetase produced by *Streptomyces tsusimaensis* (Jaitzig et al. 2013).

Data of this study show that the cultivation temperature had a significant effect on enniatin production. Lowering the temperature causes decelerated cell growth due to lower metabolic activity, which is a common method to avoid misfolding and aggregation of heterologously expressed proteins (Schein 1989; Vasina and Baneyx 1997). To compensate for the downregulated metabolism at 18 °C, the cultivation duration was doubled, enabling a prolonged and thus increased enniatin production.

In addition, feeding substrates which are building blocks of the enniatin structure boosted enniatin synthesis. Concentrations of 5 mm of l-Val and d-Hiv are sufficient for low expression of the cyclodepsipeptide. Higher feeding amounts of l-Val could promote feedback reactions, which might negatively influence the l-Val biosynthesis regulation as observed for *E. coli* (Park et al. 2011). Supplementation with 10 mm up to 15 mm d-Hiv could also result in oxidation of dispensable d-Hiv through unspecific dehydrogenases from pyruvate and amino acid metabolism, particularly those involved in l-Val, l-Leu and l-Ile biosynthesis (Massey et al. 1976). Excessive addition of l-Val apparently is detrimental and can cause overflow metabolism in the host organism *B. subtilis* as well as limiting of l-Ile biosynthesis (Castillon et al. 2011; Felice et al. 1977; Leavitt and Umbarger 1962).

For multi-copy expression of *esyn*, higher concentrations of d-Hiv and l-Val might be required to saturate effective enniatin production. Our data indicate that an increased concentration of the inductor molecule acetoin results in lower cell densities revealing nearly the same amounts of enniatin (data not shown). Reduced cell growth could be caused by higher acetoin concentrations which lead to accumulation of toxic diacetyl, catalyzed by non-enzymatic oxidations (López et al. 1975). Therefore, induction of ESYN expression at 0.1 % acetoin seems to be most advantageous with regard to cell-growth, protein synthesis and coupled enniatin production (Fig. S6).

The negative effect caused by the deletion of the autolysis gene *lytC* and the sporulation regulator gene *spoIIGA* (Kabisch et al. 2013b) on the *esyn*-expressing strain *B. subtilis* BsSZ8 was surprising. It could be speculated that the *spoIIGA* mutation has an effect on the secretion capacity of transporters for secondary metabolites. It is interesting to note that the ABC transporters OppDF, YtrBE and EcsA are specifically induced during the sporulation process (Leskelä et al. 1999; Perego and Hoch 1996; Yoshida et al. 2000). For YtrBE, a function in the uptake of the inductor acetoin has been suggested. Thus, it is conceivable that a lower intracellular inductor concentration is caused by a potential downregulation of YtrBE in a *spoIIGA* mutant. Further studies will be required to elucidate the influence of this genetic background on enniatin production in *B. subtilis*.

However, an additional deletion of the NRPS cluster encoding the surfactin synthetase in strain BsSZ10 complements for above losses in production yields and results in an enhanced enniatin production. On the one hand, the biosynthesis of surfactin relies for a considerable part on the availability of l-Val as a precursor and the capacity for posttranslational modification with Sfp, which could decrease the ESYN productivity. Therefore, a deletion of the *srfA*-cluster might lead to an accumulation of the resources available for enniatin synthesis. On the other hand, this cluster contains a beneficial type II thioesterase SrfAD for regeneration of misprimed nonribosomal peptide synthetase (Schwarzer et al. 2002). Consequently, a co-expression of this type II thioesterase could be advantageous also for the enniatin production. We have addressed not only the gene inactivation of the surfactin-relevant cluster but also the *pksX* cluster of the mixed PKS/NRPS secondary metabolite bacillaene (BsSZ12). In this case, the secondary metabolite was identified by means of ESI-mass spectrometry but no high expression level of the synthetase was detected on the protein level in SDS gels (Fig. S7). The slightly negative effect on enniatin concentration after inactivation of the *pksX* cluster was quite unexpected (Fig. 4a) and suggests that its inactivation has no direct influence on the availability of precursors or resources of enniatin production.

**Fig. 6:**
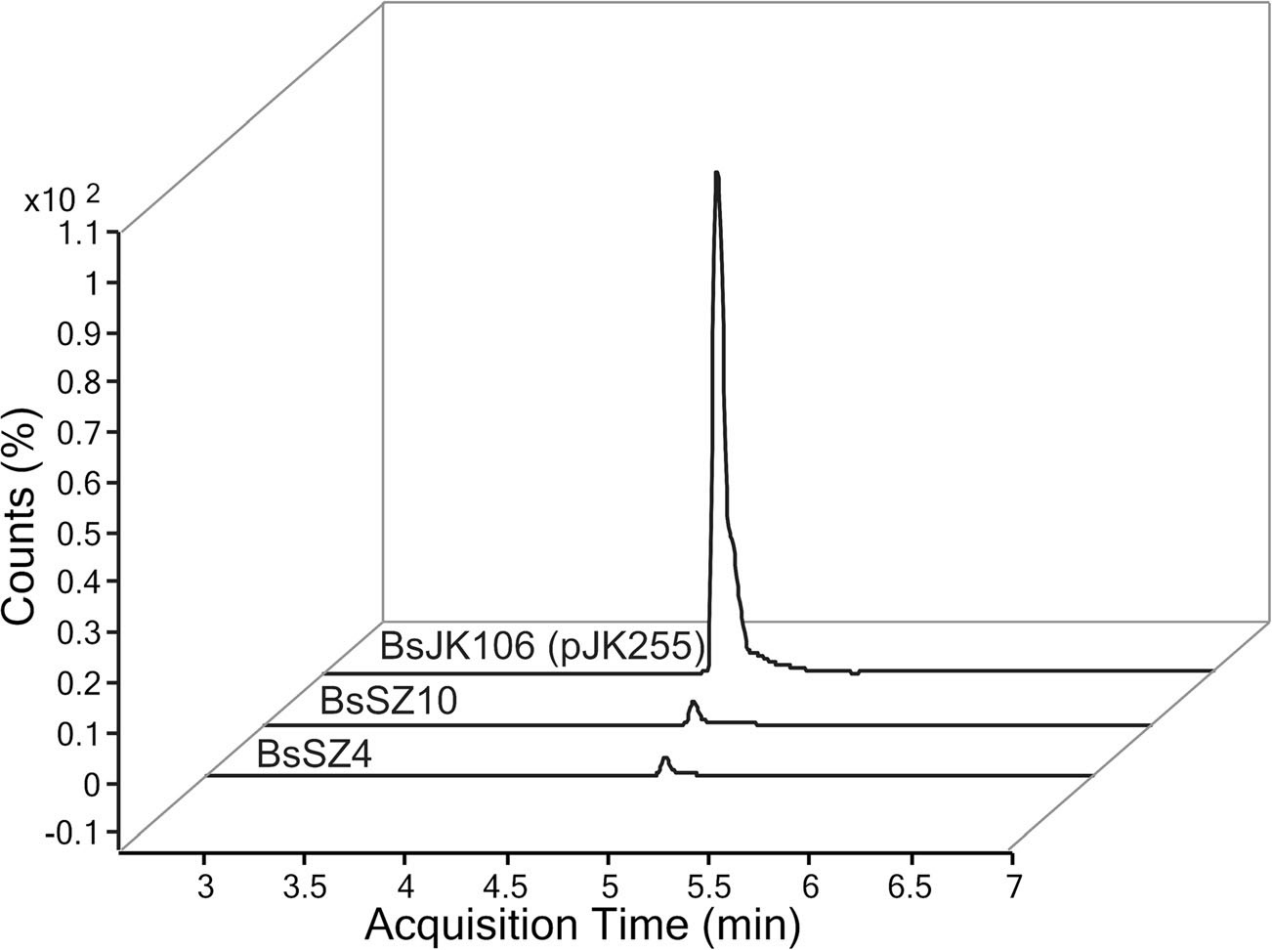
Comparison of the overall optimization of enniatin production in the heterologous producer *B. subtilis*. Quantitative HPLC-MRM mass spectrometric analysis of crude culture supernatant extracts of BsSZ4 and BsSZ10 as well as BsJK106 shows enhanced enniatin production as a result of a combination of cultivation optimization, genetic modification and high-copy expression of heterologously expressed ESYN

The highest yield of enniatin secreted into the supernatant was found for strain BsJK106 with the *esyn*-encoding multi-copy plasmid pJK255. This optimized host vector system, which originates from strain BsSZ12 and comprises gene deletions of the *srfA* and *pksX* clusters, is based on the high-copy plasmid pMSE3. In comparison to other plasmids tested, plasmid pMSE3 is distinguished (Fig. S8) by structural and segregational stability (Silbersack et al. 2006; Swinfield et al. 1991) with ∼200–250 copies per cell. Despite the metabolic burden of replication and protein synthesis by this high-copy plasmid number, as indicated by lower cell densities, enniatin synthesis significantly increased by ∼50 % (Fig. 5). It could be concluded that the deletion of energy- and metabolic precursor-consuming clusters maybe useful for multi-copy expression of ESYN in strain BsJK106. The ultimate strain of this study, BsJK106 (pJK255), produces 1.1 mg/L enniatin (Fig. 6). We assume that there is further potential for optimization of the secretory enniatin production in *B. subtilis*. This could include classical strain evolvement by mutagenesis as well as the development of suitable fed-batch cultivations including the adjustment of feeding or synthesis of the building blocks d-Hiv and l-Val. First additional optimization steps by overexpressing the appropriate transcription factors AcoR and SigL of the *acoA* promoter resulted in an almost complete collapse of enniatin production and cell growth (data not shown). Thus, a balanced ratio of gene copies, transcription and translation regulation as well as concentrations of precursors will be required. For the generation of an autonomous *B. subtilis* strain producing enniatin without additional d-Hiv feeding, exploitation of the gene coding for the d-hydroxyisovalerate dehydrogenase of *F. oxysporum* (Lee et al. 1992) could be considered by chromosomal integration into strain BsJK106.

To our knowledge, this is the first study to examine the heterologous expression of a NRPS of eukaryotic origin in the Gram-positive bacterial production host *B. subtilis*. The approach is considered to have a promising potential being applicable to generate enniatin easily in an economic continuous fermentation process since the entire cyclodepsipeptide is secreted into the medium. Furthermore, this heterologous production of enniatin can also be used for precursor-directed biosynthesis (Feifel et al. 2007; Krause et al. 2001; Matthes et al. 2012; Müller et al. 2009) of new interesting derivatives providing additional or improved bioactivity by feeding and incorporation of α-hydroxyl carboxylic acid derivatives.

## Electronic supplementary material

Below is the link to the electronic supplementary material.

ESM 1 (PDF 2087 kb)
