# Supplementary material for: Bacillus subtilis as heterologous host for the secretory production of the non-ribosomal cyclodepsipeptide enniatin
Source: Appl Microbiol Biotechnol. 2014 Nov 15;99(2):681–91. doi: 10.1007/s00253-014-6199-0 (PMC4306738; doi:10.1007/s00253-014-6199-0)
Supplement: Supplementary file 1 — (PDF 2087 kb) [file 253_2014_6199_MOESM1_ESM.pdf]

Supplementary Materials for

"Bacillus subtilis as heterologous host for the secretory production of the non-ribosomal cyclodepsipeptide enniatin"

Sophia Zobel[1]*, Jana Kumpfmüller[2]*, Roderich D. Süssmuth[1§], Thomas Schweder[2§]

[1] Institut für Chemie, Technische Universität Berlin, Strasse des 17. Juni 124, 10623 Berlin, Germany

Tel. +49-(0)30-314-78774, Fax. +49-(0)30-314-79651

Email: roderich.suessmuth@tu-berlin.de

[2] Institut für Pharmazie, Ernst-Moritz-Arndt-Universität, Felix-Hausdorff-Strasse. 3, 17489 Greifswald, Germany

Tel.: +49 (0)3834 86-4212, Fax: +49 (0)3834 86-4238

Email: schweder@uni-greifswald.de

This PDF file includes:
Materials and Methods
Table S1
Figures S1 to S8
References

Materials and methods

Construction of plasmids

The plasmid for chromosomal integration of the *esyn*-gene was constructed using a modified protocol of the Red/ET technology (Gene Bridges). First a 546 bp region representing the 3´-region of *esyn* was amplified from fosmid F9D10 (Fosmid library from *Fusarium oxysporum* ETH 1536, LGC Genomics GmbH, Berlin, Germany, CopyControl^{TM} Fosmid Library Production Kit, pCC1FOS^{TM}) using the oligonucleotides 5323 and 5330 (Tab. S1). The PCR product was cloned into pAMY-SSS, cut with *Xho*I, in front of the T7 terminator sequence via a "sequence and ligation-independent cloning" (SLIC) method (Li and Elledge 2007). This plasmid was then cut with *Stu*I and the 694 bp 5'-region of *esyn*, amplified with the primers 5331 and 5328, was then integrated via SLIC. In the subsequent step this plasmid was linearized with *Pme*I and the *acoA*-promoter, amplified from *B. subtilis* 168 with the primers 5329 and 5305, was also integrated via SLIC. The resulting plasmid now contained a P_{acoA}-*esyn* 5'-*Stu*I-3'-*esyn*-T_{T7}-cassette (P stands for promoter and T for terminator). The restriction of this plasmid with *Stu*I led to a linear product with homologous regions to the *esyn*-gene allowing recombination with the fosmid F9D10 via Red/ET according to the manufacturer's protocol. The resulting plasmid was named pJK166. The plasmid pJK166 was used to chromosomally integrate the *esyn* gene cluster under control of the *acoA* promoter into the *amyE l*ocus, resulting in *B. subtilis* JK3.

For multi-copy expression, the high-copy *E. coli*/*B. subtilis* shuttle vector pMSE3 was used (Silbersack et al. 2006). The P_{acoA}-*esyn*-T_{T7}-cassette, obtained from pJK166 and digested with *Xba*I/*Bst*Z17I, was ligated into pMSE3 after cutting this plasmid with *Xba*I and *Sma*I, resulting in pJK255.

In order to improve the transformation efficiency in naturally competent *B. subtilis* cells, an IPTG-inducible copy of the gene for the competence regulator ComS was chromosomally integrated (Liu et al. 1996). For this purpose, plasmid pBB1366 was cut with *Bam*HI and *Eco*RI and a cassette containing the *comS* gene regulated by the *spac*-promoter and *lacI* repressor was inserted after restriction of pKE27 in the same manner. Both fragments were ligated to give plasmid pJK45.

In order to combine the optimized technique for rapid genome modifications of *B. subtilis* (Kumpfmüller et al. 2013) with the second *comS* copy, a plasmid for combined chromosomal integration of the *lacI*-P_{spac}-*comS*-cassette and the *xylR*-P_{xylA}-*cre*-cassette was constructed. Therefore, a PCR product resulting from the primer pair 5381 and 5382 and pDGICZ as template was integrated via the SLIC method into plasmid pBB1366, which was cut with *Bss*HII. Subsequently, this plasmid was digested with *Bam*HI and *Eco*RI and the *xylA*-promoter gene, obtained from pX (Kim et al. 1996) by using the primers 5394 and 5395, was inserted via the SLIC method. The *cre* gene, amplified from pDGICZ with the oligonucleotides 5396 and 5397 was inserted into the *Bam*HI linearized plasmid by using the same method. The resulting plasmid (pJK195) was then digested with *Mfe*I. Via the SLIC method the *comS* cassette from pJK45, amplified by with the primers 5398 and 5399, was inserted resulting in the final plasmid pJK196.

As *B. subtilis* 168 contains a frame shift in the *sfp* gene responsible for NRPS activation it was necessary to substitute the mutated gene by a functional version which was obtained from *B. subtilis* 6051HGW (NCBI CP003329) (Kabisch et al. 2013b). A 2891-bp DNA fragment was amplified comprising the region surrounding the *sfp* gene using the oligonucleotides 5026 and 5027. The fragment was integrated via the SLIC method into plasmid pAMYSSE which was cut previously with *Nar*I and *Eco*RI to give plasmid pJK63. Hereafter, this plasmid was cut with *Aat*II and *Not*I. The downstream region of the *sfp* gene containing *yckI* was amplified from the same host using the primer pair 5028 and 5029 and was integrated into pJK63 yielding plasmid pJK64. Within this plasmid, the six-site-spec^{R}-cassette (SSS-cassette) was substituted by the improved *lox*-SSS-cassette (Kumpfmüller et al. 2013) by digest of pJK64 (*Eco*RI/*Sna*BI) and pJET-*lox*-SSS (*Mfe*I/*Eco*RV). The ligation of the respective fragments resulted in plasmid pJK64a.

In order to improve the stability of heterologous DNA and to enhance the transformation efficiency of large plasmids the restriction and modification (RM) system of *B. subtilis* was deleted according to (Choi et al. 2009). For this purpose, the corresponding 5'-region was amplified using the primers 5421 and 5422 and cut with *Msc*I and *Bam*HI. The amplified fragment was integrated into plasmid pAMY-lox-SSS, cut with *Msc*I and *Bam*HI, via the SLIC method. In a following SLIC reaction the 3´-region (amplified from *B. subtilis* using the oligonucleotides 5423 and 5424) was integrated into the plasmid obtained from digest with *Spe*I and *Aat*II to give plasmid pJK226.

Following the optimization of *B. subtilis* 6051HGW as expression host (Kabisch et al. 2013a) the genes *lytC* and *spoIIGA* involved in cell lysis and sporulation were also deleted. Therefore, the *lox*-SSS-cassette obtained from pJET-*lox*-SSS by digest with *Stu*I was ligated into plasmid pLytC, cut with *Bst*Z17I, to give pJK205 and integrated into plasmid pSpoIIGA, cut with *Bst*Z17I, to give pJK209, thereby replacing the respective six-site-marker-cassettes.

To reduce the metabolic burden of the expression strain, selected host-own NRPS and PKS gene clusters were deleted. For this purpose gene deletion plasmids for *srfA* (deleted *srfAA*-*srfAD*; approximately 26 kb of the surfactin biosynthesis cluster) and *pksX* (deleted *pksA*-*pksR*; approximately 76 kb of bacillaene biosynthesis cluster) were constructed as follows: The *srfA* 5´-region with the reconstituted *comS* gene (under the control of the *srfAA*-promoter) was amplified from pKE19 using the oligonucleotides 5173 and 5174, and integrated into the linearized plasmid pAMY-Kan after digestion with *Aat*II/*Spe*I by SLIC. The resulting plasmid was then digested with *Sna*BI/*Xho*I and ligated with the *sfrA* 3´-region obtained from *B. subtilis* after amplification using the primers 5180 and 5182 and cut with *Sna*BI/*Xho*I. The resulting plasmid (designated pJK93) was then digested with *Eco*RI and *Xba*I to replace the kanamycin resistance cassette by the *lox*-SSS-cassette obtained from pJET-lox-SSS (Kumpfmüller et al. 2013) after restriction with *Mfe*I and *Xba*I to finally give the *srfA* deletion plasmid pJK191.

The 5´-region of the *pksX* cluster was amplified from *B. subtilis* using primers 5367 and 5368 and integrated via SLIC into plasmid pSigL after its restriction with *Nar*I and *Eco*RI. The resulting plasmid

was then cut with *Not*I and *Aat*II and the 3´-region of this operon (amplified with the primer pair 5386 and 5387) was integrated using the same method. In a subsequent step, the six-site-ery^{R}-cassette was removed by digestion with *Eco*RI and *Xba*I and substituted by the *lox*-SSS-cassette after restriction of pJET-lox-SSS with *Mfe*I and *Xba*I yielding the *pksX* deletion plasmid pJK179.

For removal of the *comS-cre*-cassette by substitution with a spectinomycin marker (*spec*^{R}), plasmid pJK196 was digested with *Asc*I and ligated with a *spec*^{R} cassette, obtained from pAMY-SSS by amplification with the oligonucleotides 5417 and 5418 and digest with *Asc*I, to give pJK210. To enhance the integration accuracy of this plasmid, the *sacA* landing pad was replaced by a small *lacI* 3´-region. Therefore, this part was amplified from *B. subtilis* using the primers 5468 and 5470 and integrated into pJK210 after digest with *Dra*I via the SLIC method to give plasmid pJK256.

Construction of strains

For chromosomal integrations, *B. subtilis* cells were made naturally competent using the protocol of Kumpfmüller et al. (2013). For *comS*-induction 100 µM IPTG was added when the cells were diluted. To remove the antibiotic selection marker after successful chromosomal integration two methods based on site-specific recombination were used: i) recombination of flanking six-sites with a plasmid-coded beta-resolvase using a protocol previously described (Kabisch et al. 2013b), ii) recombination of flanking *lox* sites with chromosomal integrated *cre* as previously reported by Kumpfmüller et al. (2013).

For single-copy expression, *B. subtilis* 168 was transformed with linearized pJK45 to give *B. subtilis* JK3. In a first attempt, we initially reconstituted the *sfp* defect by chromosomal integration of pJK64 in *B. subtilis* JK3 followed by marker removal via method i). This methods leaves an approx. 450 bp "six-site" sequence in the chromosome (Sanchez et al. 2007). The resulting strain should be used for chromosomal integration of the P_{acoA}-*esyn*-T_{T7} operon (via pJK166) into the *amyE* gene locus, thereby destroying the α-amylase activity. However, due to an unwanted crossover event between the six-site localized in the genome and the six-sites flanking the marker cassette in the plasmid pJK166 no amylase-negative colonies could be obtained. To overcome this problem the pJK166 plasmid was directly integrated into *B. subtilis* JK3 to give *B. subtilis* SZ1. This way, almost all colony forming units obtained showed an amylase-negative phenotype indicating that the *esyn* operon was integrated successfully. Removal of the selection marker (method i) resulted in *B. subtilis* SZ2. This strain was then transformed with the pJK64 plasmid to reconstitute the PPTase activity (Sfp). Successful integration could be verified via hemolytic activity on sheep blood agar plates due to surfactin production. After removal of the selection marker using the same technique *B. subtilis* SZ4 was obtained.

For further modification of the expression strain *B. subtilis* SZ4, plasmid pJK196 was chromosomally integrated in order to use the *cre*-*lox*-system for marker removal (method ii). The obtained strain, *B. subtilis* SZ5, was then transformed with pJK205 to give *B. subtilis* SZ6, followed by the chromosomal integration of plasmid pJK209 yielding *B. subtilis* SZ7. In a next step, this strain was

transformed with plasmid pJK191 to give *B. subtilis* SZ9. Hereafter, plasmid pJK179 was chromosomally integrated resulting in *B. subtilis* SZ11.

In order to remove the *comS*-*cre*-cassette from *B. subtilis* SZ7, *B. subtilis* SZ9 and *B. subtilis* SZ11, these strains were transformed with plasmid pJK210 to give *B. subtilis* SZ8, *B. subtilis* SZ10 and *B. subtilis* SZ12.

For multi-copy expression, *B. subtilis* 168 was transformed with linearized pJK196 to give *B. subtilis* JK13. The following strains were constructed using the *lox*-six-site-marker cassette and the chromosomally integrated *cre* for marker removal (method ii). In a first step, *B. subtilis* JK13 was transformed with linearized plasmids pJK64a to give *B. subtilis* JK28. This strain was transformed with linearized plasmid pJK226 yielding in *B. subtilis* JK46. Chromosomal integration of plasmid pJK191 resulted in *B. subtilis* JK75. In a next step, this strain was transformed with plasmid pJK179 to give *B. subtilis* JK76. Successive integration of plasmids pJK205 and pJK209 yielded *B. subtilis* JK77 and *B. subtilis* JK78. For *amyE* gene inactivation (like single-copy strains BsSZ4–12) plasmid pAMY-lox-SSS was chromosomally integrated to give *B. subtilis* JK105. In a final step, this strain was transformed with pJK256, thereby replacing the *comS*-*cre*-cassette with the spectinomycin marker, yielding in *B. subtilis* JK106.

Table S1 Oligonucleotide primers used in this study. Spec^R: spectinomycin resistance cassette; Zeo^R: zeocin resistance cassette; RM: restriction and modification system

Name	Sequence 5' → 3'	Usage
5026	ACGCGGGGAGGCAGACAAGGTATAGGGCG GCGCCCAGGCTTCCATCTATCCGTC	Forward primer for *sfp*-5´-region
5027	CTAAAATTGGTTATGCACGACTCTACGAATT CGAATGCAAGGGTTTGCCAAC	Reverse primer for *sfp*-5´-region
5028	TAGTATTAGTAATTATCAGAATTGATCTGCG GCCGCGTTGTCAGGCCTGTGCTTCG	Forward primer for *sfp*-3`-region
5029	CGCGCACATTTCCCCGAAAAGTGCCACCTG ACGTCGTCCAGCATGTCATCGAACG	Reverse primer for *sfp*-3`-region
5173	GGCCCCAAGGGGTTATGCTATCTAGATCGA CTAGTGGCCGCCTGAATTG	Reverse primer for *srfA*-5´-*comS*-region
5174	CGCGCACATTTCCCCGAAAAGTGCCACCTG ACGTCCCGCCGAAGGTTGAATA	Forward primer for *srfA*-5´-*comS*-region
5180	CGCCTACGTATAGGTGCTCTGCCAGCC	Forward primer for *srfA*-3`-region
5182	GGATCCTCGAGAATTCATCCGATCATTCAAC CGTGATCAAAAGCGG	Reverse primer for *srfA*-3`-region
5305	GCATCACGTATTCAGCTGGATCCTCGAGGGT TTATTCAGTCAAACGATG	Forward primer for *acoA*-promoter
5323	GTTATGCTAACTAGTATCGATATCGAATTCT CGAGCTACAAAGCCTCGTTCAAAC	Forward primer for *esyn*-3`-region
5328	GCAGCATCACGTATTCAGCTGGATCCTCGA GGGTTTAAACCATATGTCACTCCACACCCCA AG	Forward primer for *esyn*-5´-region
5329	GTCACTTGGGGTGTGGAGTGACATATGGTTT CCTCCTTCTATTTAGGGTTC	Reverse primer for *acoA*-promoter
5330	GCAGCATCACGTATTCAGCTGGATCCTCGA GGCCTGTGCCCTTGTCCTAGC	Reverse primer for *esyn*-3`-region
5331	CGGATTCCTTGGCTAGGACAAGGGCACAGG CCTTCCAAAACTCAACAGCAC	Reverse primer for *esyn*-5´-region

Table S1 continued

5367	CGCGGGGAGGCAGACAAGGTATAGGGCGG CGCCGAATGCCGCTCAACGCCTCG	Forward primer for *pksX*-5´-region
5368	CTAAAATTGGTTATGCACGACTCTACGAATT CGTAACAAGAAAAAAATGAGAG	Reverse primer for *pksX*-5´-region
5381	CGCGGGCTTCAACGGGCTGGACGATTTTGG CGCGCCTCAGTCCTGCTCCTCGGCC	Forward primer for *Zeo*^{R} cassette
5382	CAGTGGCAGTCAATGGTCGGATGGGGCGCG CCGAATTCTACACAGCCCAGTCC	Reverse primer for *spac*-promoter
5386	GTATAGCATACATTATACGAACGGTAGGCC TCTAGATGAATTGGTGAAGCGCTG	Forward primer for *pksX*-3`-region
5387	CGCGCACATTTCCCCGAAAAGTGCCACCTG ACGTCTTCGATGCTGTTCTTGCTC	Reverse primer for *pksX*-3`-region
5394	GAGTAACACATATAAAAAGCCATATCAAGG ATCCCATTTCCCCCTTTG	Forward primer for *xylA*-promoter
5395	GTCAATGGTCGGATGGGGCGCGCCGAATTC AATTGACCAACTGGTAATGGTAGC	Reverse primer for *xylR* gene
5396	GAGTAACACATATAAAAAGCCATATCAAGG ATCCTAATCGCCATCTTCCAGC	Forward primer for *cre* gene
5397	CTAAAAATCAAAGGGGGAAATGGGATCCAT GTCCAATTTACTGACCG	Reverse primer for *cre* gene
5398	GCGCCGGTCGCTACCATTACCAGTTGGTCGT TCTACATCCAGAACAACC	Forward primer for *spac*-promoter
5399	GGTCGGATGGGGCGCGCCGAATTCAATTCC AACTGGTAATGGTAGCG	Reverse primer for *lacI* gene
5417	CAACGGGCTGGACGATTTTGGCGCGCCTAA TTTCACAAGAGGACG	Reverse primer for Spec^R cassette
5418	CAATGGTCGGATGGGGCGCGCCAATCTAGG GTAAGTAAATTGAG	Forward primer for Spec^R cassette

Table S1 continued

5421	CGACAGGAGCACGATCATGCGCACCCGTGG CCAATCCGTCAAATCGCAC	Forward primer for RM-5´-region
5422	ATGCTATACGAACGGTAGGCCTCGAGGATC CAGCAACAATTTGGTC	Reverse primer for RM-5´-region
5423	GAACGGTAGGCCTCTAGAGTCAGATCGATA CTAGTGAGATTGCTGACAGAAC	Forward primer for RM-3`-region
5424	AGCAATTATTACCTCCACGGGGAGAGCCTG ACGTCAATGATTTAGACGGAGC	Reverse primer for RM-3`-region
5468	ATTTTATTGCAATAACAGGTGCTTACTTTTA AACCAGCGTGGACCGC	Forward primer for *lacI*-3`-region
5470	CAAAAAGGATCTTCACCTAGATCCTTTTAAA TGGTAGCGACCGGCGCTC	Reverse primer for *lacI*-3`-region

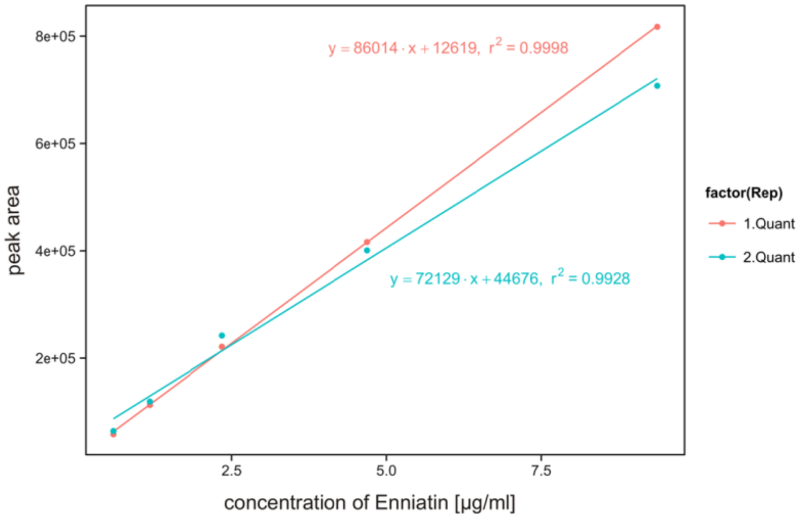

Fig S1 Calibration curves of enniatin for quantification with an external enniatin standard. N=2

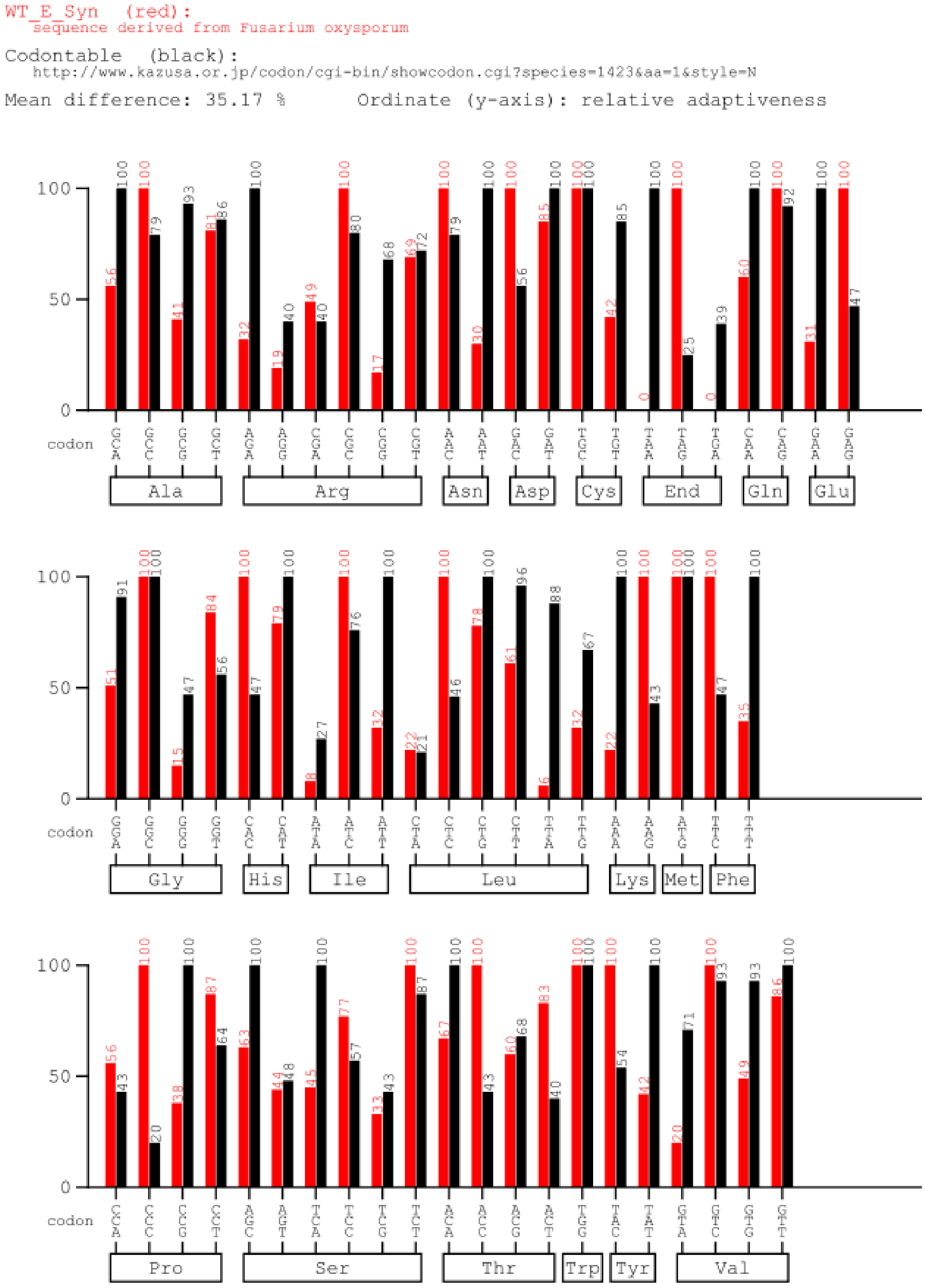

Fig. S2 Overview of codon usage in comparison to *esyn* sequence between *F. oxysporum* (red bars) and *B. subtilis* (black bars). Depicted is the relative adaptiveness of the codon usage to the *esyn*-gene sequence.

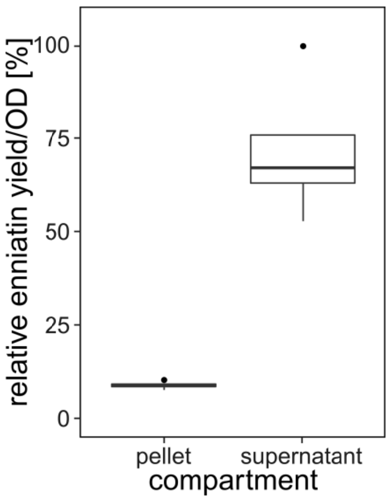

Fig. S3 Comparison of the enniatin concentration in the biomass and supernatant. The biomass and the supernatant of *B. subtilis* BsSZ4 cultures were extracted and the enniatin level was quantified by HPLC-ESI-MRM mass spectrometry. N=4

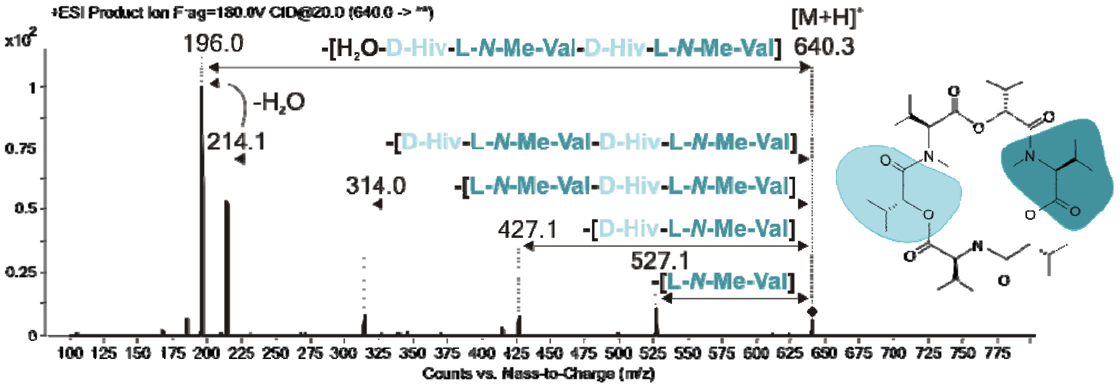

Fig. S4 HPLC-ESI-MS^2 analysis of heterologously produced enniatin. For identification of enniatin synthesized by *B. subtilis* the molecular ion ([M+H]*= 640.3 Da) was fragmented and characteristic fragments assigned.

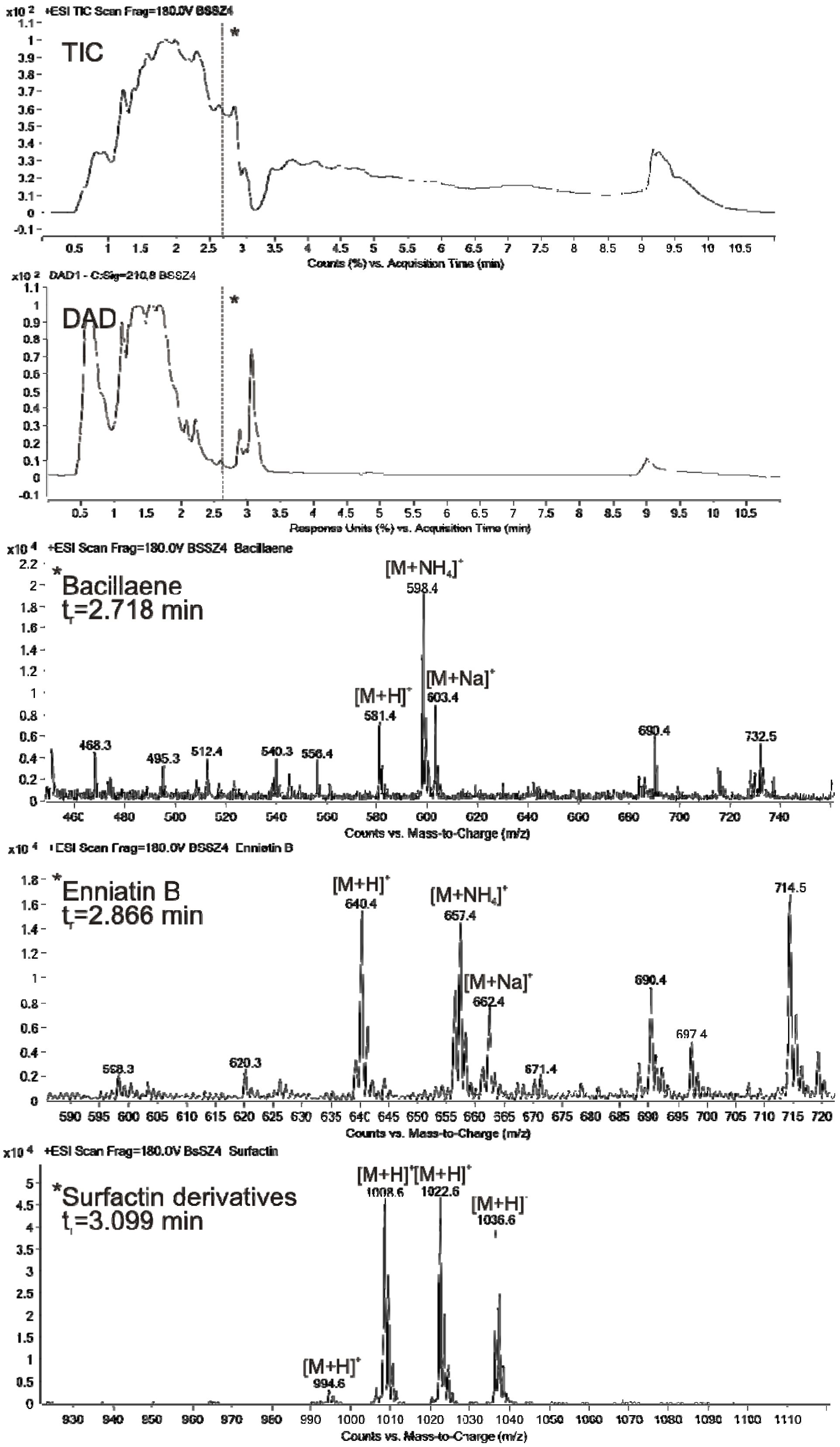

Fig S5 HPLC-ESI-MS^2 scan analysis of crude BsSZ4 extract. Detection of secondary metabolites synthesized by the NRPS and PKS/NRPS biosynthesis machinery of *B. subtilis*. Evidence for presence of the lipopeptide surfactin and the PKS/NRPS hybrid bacillaene led to the systematic inactivation of relevant gene clusters in order to enhance enniatin production. Depicted is the total ion chromatogram (TIC) of measured extract of BsSZ4 as well as the diod array detector (DAD) chromatogram with a wavelength of 210 nm and the according spectras for bacillaene, enniatin and surfactin derivatives.

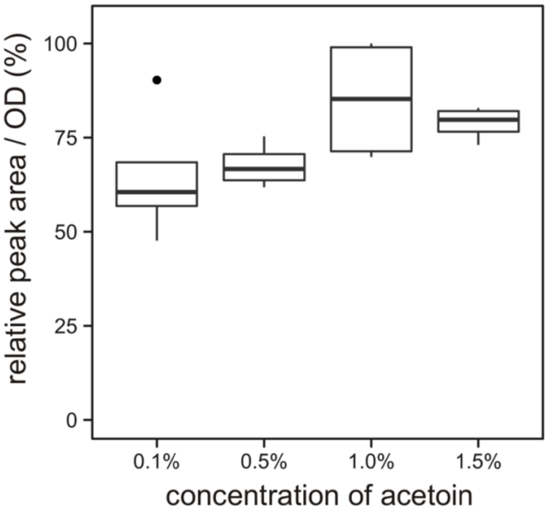

Fig. S6 Relative enniatin production by variation of acetoin inductor concentration. Higher concentrations of acetoin (0.5%, 1.0% and 1.5%) in the cultivation medium result in higher relative enniatin production measured by the MRM experiment (ESI-mass spectrometry). Simultaneously the cultures reached lower cell densities presumably because of toxic side products through non enzymatic oxidation of acetoin. N=4

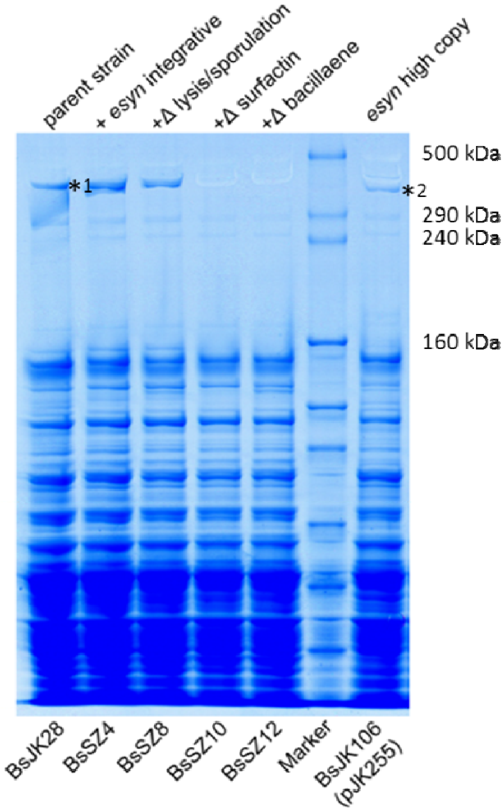

Fig. S7 1D-protein analysis of the enniatin producing *B. subtilis* strains. Coomassie Blue-stained NuPage® 3–8% Tris-Acetate Gel (Life Technologies) showing the intracellular, soluble protein fraction of engineered *B. subtilis* strains after cultivation at 18 °C for 48 h in 20 mL SB medium (0,1% acetoin, without D-Hiv). A culture volume according to 20 OD-units was harvested and cells were mechanically disrupted via RiboLyser (45 s at 4.5 m/s). After centrifugation (15 min, 13 000 rpm, twice) 20 µg of the soluble protein fraction was loaded on the gel

according to the manufacturer's protocol. BsJK28: parental strain; BsSZ4: + *esyn* (single copy); BsSZ8: with a *lytC/spoIIGA* inactivation; BsSZ10: deletion of the surfactin cluster; BsSZ12: with an additional inactivation of bacillaene synthesis. BsJK106 (pJK255): high copy *esyn* expression. *1: SrfAA (402 kDa) + SrfAB (401 kDa); *2: ESYN (347 kDa)

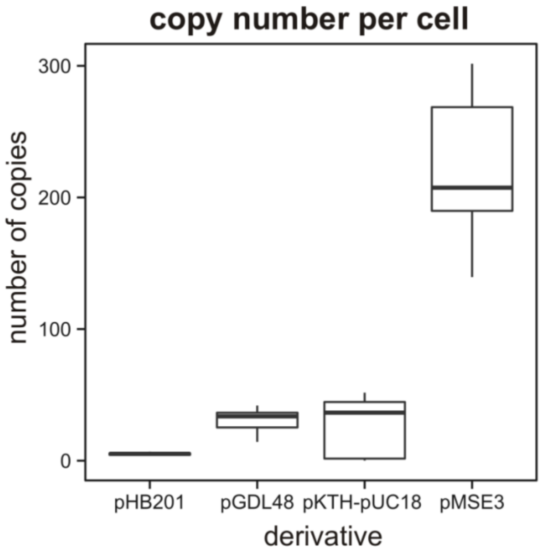

Fig. S8 Comparison of different plasmids with regard to plasmid stability and copy numbers per cell in *B. subtilis*. Determination of numbers of plasmid copies revealed 200-250 for the pMSE3 backbone that was used for multi-copy expression of *esyn* in BsJK106. N=6

References

Choi S-K, Park S-Y, Kim R, Kim S-B, Lee C-H, Kim JF, Park S-H (2009) Identification of a Polymyxin Synthetase Gene Cluster of *Paenibacillus polymyxa* and heterologous expression of the gene in *Bacillus subtilis*. J Bacteriol 191:3350–3358

Kabisch J, Pratzka I, Meyer H, Albrecht D, Lalk M, Ehrenreich A, Schweder T (2013a) Metabolic engineering of *Bacillus subtilis* for growth on overflow metabolites. Microb Cell Factories 12:72

Kabisch J, Thürmer A, Hübel T, Popper L, Daniel R, Schweder T (2013b) Characterization and optimization of *Bacillus subtilis* ATCC 6051 as an expression host. J Biotechnol 163:97–104

Kim L, Mogk A, Schumann W (1996) A xylose-inducible *Bacillus subtilis* integration vector and its application. Gene 181:71–76

Kumpfmüller J, Kabisch J, Schweder T (2013) An optimized technique for rapid genome modifications of *Bacillus subtilis*. J Microbiol Methods 95:350–352

Li MZ, Elledge SJ (2007) Harnessing homologous recombination in vitro to generate recombinant DNA via SLIC. Nat Methods 4:251–256

Liu L, Nakano MM, Lee OH, Zuber P (1996) Plasmid-amplified comS enhances genetic competence and suppresses sinR in *Bacillus subtilis*. J Bacteriol 178:5144–5152

Sanchez H, Cozar MC, Martinez-Jimenez MI (2007) Targeting the *Bacillus subtilis* genome: An efficient and clean method for gene disruption. J Microbiol Methods 70:389–394.

Silbersack J, Jürgen B, Hecker M, Schneidinger B, Schmuck R, Schweder T (2006) An acetoin-regulated expression system of *Bacillus subtilis*. Appl Microbiol Biotechnol 73:895–903
